# Crenarchaeal Biofilm Formation under Extreme Conditions

**DOI:** 10.1371/journal.pone.0014104

**Published:** 2010-11-24

**Authors:** Andrea Koerdt, Julia Gödeke, Jürgen Berger, Kai M. Thormann, Sonja-Verena Albers

**Affiliations:** 1 Molecular Biology of Archaea, Max Planck Institute for Terrestrial Microbiology, Marburg, Germany; 2 Department of Ecophysiology, Max Planck Institute for Terrestrial Microbiology, Marburg, Germany; 3 Electron Microscopy, Max Planck Institute for Developmental Biology, Tübingen, Germany; University of Münster, Germany

## Abstract

**Background:**

Biofilm formation has been studied in much detail for a variety of bacterial species, as it plays a major role in the pathogenicity of bacteria. However, only limited information is available for the development of archaeal communities that are frequently found in many natural environments.

**Methodology:**

We have analyzed biofilm formation in three closely related hyperthermophilic crenarchaeotes: *Sulfolobus acidocaldarius*, *S. solfataricus* and *S. tokodaii*. We established a microtitre plate assay adapted to high temperatures to determine how pH and temperature influence biofilm formation in these organisms. Biofilm analysis by confocal laser scanning microscopy demonstrated that the three strains form very different communities ranging from simple carpet-like structures in *S. solfataricus* to high density tower-like structures in *S. acidocaldarius* in static systems. Lectin staining indicated that all three strains produced extracellular polysaccharides containing glucose, galactose, mannose and N-acetylglucosamine once biofilm formation was initiated. While flagella mutants had no phenotype in two days old static biofilms of *S. solfataricus*, a UV-induced pili deletion mutant showed decreased attachment of cells.

**Conclusion:**

The study gives first insights into formation and development of crenarchaeal biofilms in extreme environments.

## Introduction

In nature, most microbes are assumed to exist predominantly in surface-associated communities, encased in a self-produced matrix, termed biofilms [1], [2], [3]. Thus, the formation of biofilms reflects the native growth conditions for most microbial species. Cells within biofilms differ substantially from their planktonic counterparts, particularly with regard to an increased resistance towards numerous environmental perturbations. Thus, the mechanism of biofilm formation and its importance for microbial survival in natural habitats has attracted increasing interest in recent years.

To date, studies on microbial biofilms have mainly been conducted on bacteria, in particular with regard to pathogenic species in which biofilms play an important role in disease development [4], [5]. In sharp contrast, for the archaeal domain only very limited information is available on this topic. Archaea are frequently detected in biofilm communities from many different environments [6], [7], but biofilm formation by archaea has only been sparsely studied. So far, all studies have dealt with the formation of biofilms by euryarchaeotes. The first archaeal biofilm was described for the hyperthermophilic *Thermococcus litoralis*. The *T. litoralis* biofilm developed in rich media on hydrophilic surfaces, such as polycarbonate filters, and was accompanied by mannose-type extracellular polysaccharides production [8]. *Archaeoglobus fulgidus* biofilm formation, measured as attachment to the sides of cultivation vessels, was found to be increased in response to unfavorable environmental conditions, including high metal concentrations, pH and temperature changes [9]. Upon adhesion to (a)biotic surfaces, mediated by flagella or pili, *Pyrococcus furiosus* and *Methanobacter thermoautotrophicus* formed monospecies biofilms, respectively [10], [11]. Development of *P. furiosus* and *Methanopyrus kandlerii* bi-species biofilms was shown to be established within less than 24 hours on biotic surfaces [12].However, the formation of a layered biofilm was dependent on the initial colonization of the surface by *M. thermoautotrophicus* cells to which *P. furisous* could adhere by using its flagella and establishing cell-to-cell contacts. Very recently, two distinct biofilm morphologies were described in the extremely acidophilic euryarchaeote *Ferroplasma acidarmanus* Fer1, a multilayered film forming on glass and pyrite surfaces and up to 5 mm-long filaments that were also found in natural environments [13]. Proteomic studies on these biofilms showed that 6 out of the 10 up-regulated proteins were involved in the adaptation to anaerobic growth indicating anaerobic zones in the multilayered *Ferroplasma* biofilms.

In this study, we use the crenarchaeal model organism *Sulfolobus spp.* to initiate comprehensive studies on archaeal biofilms. *Sulfolobus* species are hyperthermoacidophiles growing optimally at 70–85°C and pH 2–3 that are found worldwide in geothermically active environments such as solfataric fields. They express a variety of surface structures including flagella and type IV-like pili [14], [15], [16] which have been shown to be involved in motility and UV light-induced cell aggregation [16], [17]. A recent study indicated that flagella and pili are also essential for initial surface attachment [18]. The same study demonstrated that *Sulfolobus* can attach to a variety of surfaces including glass, mica, pyrite and gold coated carbon grids. An initiation of microcolony formation by the attached cells was observed, indicating that *Sulfolobus* may be able to develop into structured microbial communities reminiscent to that of many eubacteria.

To further assess the ability to form biofilms, *S. solfataricus*, an European isolate from Italy [19], *S. acidocaldarius*, originally isolated from Yellowstone National Park [20] and *S. tokodaii*, an isolate from Japan [21], were chosen for a comparative study. Using an adapted microtitre plate assay, the impact of multiple environmental conditions on biofilm formation by these three *Sulfolobus* species was tested. Confocal laser scanning microscopy (CLSM) was employed to study the formed microbial communities in detail. We demonstrate that all three *Sulfolobus* species develop into distinct three-dimensional communities. The adapted methods will enable further detailed studies on how archaeal biofilms are formed and how their structures develop.

## Materials and Methods

### Strains and growth conditions

*Sulfolobus solfataricus* P2 (DSM1617), *S. acidocaldarius* (DSM639), *S. tokodaii* (DSM16993), *S. solfataricus* PBL2025 [22], flagella deletion mutant Δ*flaJ* [16] and the ups pili deletion mutant Δ*upsE* [17] were grown in Brock medium at 76°C, pH adjusted to 3 using sulphuric acid, and supplemented with 0.1% w/v tryptone [20]. For biofilm formation, cultures were inoculated in standing Petri dishes and grown for 2–3 days at 76°C in a metal box which was supplemented with a small amount of water to minimize evaporation of the media. For these assays *Sulfolobus* strains were inoculated with specific starting OD_600_ of 0.03 for *S. solfataricus*, 0.01 for *S. acidocaldarius* and 0.06 for *S. tokodaii*.

### Microtitre plate assay

The assay was performed in polystyrol 96-well tissue culture plates (flat bottom cell+, Sarstedt) to screen for the efficiency of biofilm formation under different environmental conditions. To avoid evaporation of the medium, the plates were covered with a gas-permeable sealing membrane (Breathe-Easy, Diversified Biotech, Boston, USA). After two days incubation the microtitre plate was cooled down to room temperature and the OD_600_ of cell cultures from each well was measured using a luminometer (InfiniteM200, TECAN, Switzerland) at a wavelength of 600 nm. 10 µl of a 0.5% solution of crystal violet (CV) was added and incubated at room temperature for 10 minutes. Subsequently, the liquid supernatant was removed from each well and the biofilm cells attached to the well were washed with water. 100% ethanol was added to release the crystal violet from the biofilm. The absorbance of crystal violet from each well was measured at a wavelength of 570 nm. The efficiency of biofilm formation was determined by the correlation between the growth of the cells (OD_600 nm_) and the absorbance of crystal violet (OD_570 nm_).

To determine how much biomass was present as biofilm, biofilms were grown and either resuspended by prolonged vortexing to obtain the OD_600 nm_, or stained with crystal violet to obtain the corresponding OD_570 nm_ values. This relation was used to calculate the percentage of cells within the biofilm related to the total amount of cells in biofilm and planktonic cells (see Fig. S2 and Table S1).

### Confocal laser scanning microscopy (CLSM)

For CLSM images the cells were grown for three days in uncoated plastic dishes (µ-Dishes, 35 mm high; Ibidi, Martinsried). To prevent evaporation at the high incubation temperature, the lids of the dishes were closed. The medium was carefully exchanged every 24 hours to ensure aerobic growth conditions. Prior to confocal microscopy, the liquid supernatant of the biofilm, with the planktonic cells, was removed and 2 ml fresh medium was added. Images were recorded on an inverted TCS-SP5 confocal microscope (Leica, Bensheim, Germany).

DAPI (4′,6-diamidino-2-phenylindole), dissolved in water to 300 µg/ml, was used to visualize the cells of the biofilm. 6 µl of the DAPI stock solution was added to the biofilm and incubated at room temperature for at least 10 minutes. Images were taken at an excitation wavelength of 345 nm and an emission wavelength of 455 nm.

A 100 µM stock solution of 7-hydroxy-9H-1,3-dichloro-9,9-dimethylacridin-2-one (DDAO; Invitrogen, Karlsruhe, Germany), in demineralised water, was used at a final concentration of 4 µM. Incubation times varied between 20 and 300 minutes. DDAO has an excitation wavelength of 646 nm and an emission wavelength of 659 nm.

Fluorescently labelled lectins were employed to visualize the EPS (extracellular polymeric substances) of the biofilms. Before adding lectins to the biofilm, the growth medium was replaced with medium adjusted to pH 5 to ensure that binding of lectins was not inhibited by low pH. Fluorescein-conjugated concavalin A (ConA) (5 mg/ml; Invitrogen, Karlsruhe, Germany), which binds to α-mannopyranosyl and α-glucopyranosyl residues, was dissolved in 20 mM sodium bicarbonate (pH 8) to a final concentration of 10 µg/ml. Fluorescein-conjugated ConA has an excitation wavelength of 494 nm and an emission wavelength of 518 nm.

Alexa Fluor® 594-conjugated GS-II, specific for N-acetyl-D-glucosamine (lectin GS-II from *Griffonia simplicifolia*, 1 mg/ml; Invitrogen, Karlsruhe, Germany), and IB_4_, specific for α-D-galactosyl residues (isolectin GS-IB4 from *Griffonia simplicifolia* 1 mg/ml; Invitrogen, Karlsruhe, Germany), were dissolved in 100 mM Tris-HCl pH 7.4 and 0.5 mM CaCl_2_ to final concentrations of 8 µg/ml.

The Alexa Fluor-conjugated lectins, which have an excitation wavelength of 591 nm and an emission wavelength of 618 nm, were used in concert with ConA. The lectin-biofilm mixtures were incubated at room temperature for 20–30 minutes in the absence of light. After incubation, the biofilm was washed with Brock media (pH 5) to remove excess label and images were taken by CSLM. Image data obtained were processed by using the IMARIS software package (Bitplane AG, Zürich, Switzerland).

### Scanning electron microscopy

*S. acidocaldarius* was grown as a standing culture under the described biofilm conditions in Petri dishes with 30 ml brock media adjusted with 0.1% trypton together with polylysin treated glass coverslips. The cells were fixed with 2.5% glutaraldehyde and incubated for 5 min at room temperature. The coverslips were carefully removed and stored at 4°C in 24 well plates with PBS-buffer with 2,5% glutaraldehyde.

The samples were then postfixed with 1% osmium tetroxide for 1 h on ice. After washing the cells were dehydrated in ethanol and critical-point-dried from CO_2_. The samples were sputter-coated with 7 nm Au/Pd and examined at 20 kV accelerating voltage in an Hitachi S-800 field emission electron microscope.

## Results

### Adaptation of microtitre plate assay

**Figure 1:**
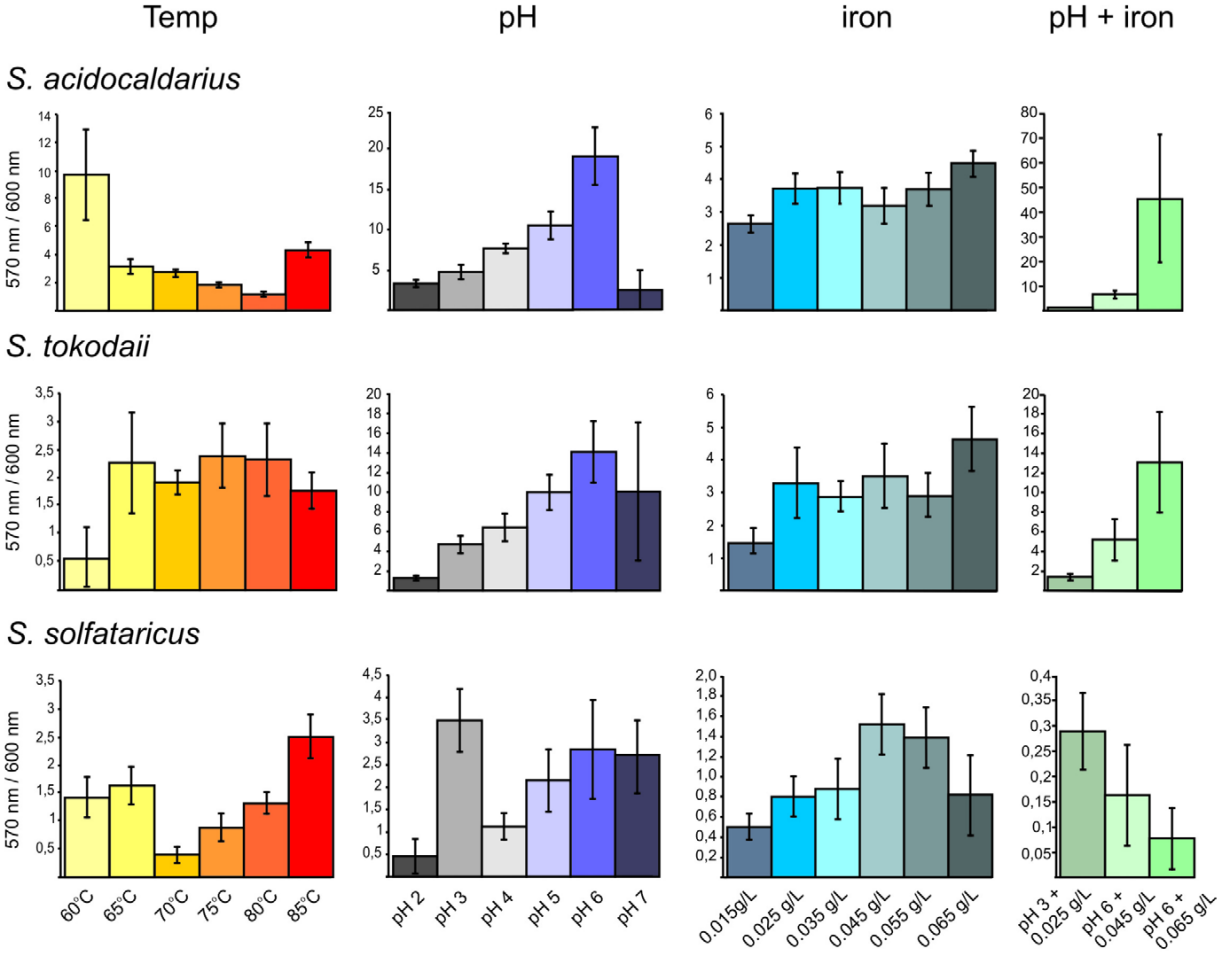
Effect of varying conditions on biofilm formation of the three *Sulfolobus* strains in microtitre plates. *S. acidocaldarius* (first row), *S. tokodaii* (second row) and *S. solfataricus* (third row) were incubated at different temperatures (first column), pH values (second column), iron concentrations (third column) and a combination of different iron concentrations and pH values (fourth column). The graphs show the correlation of the measured cristal violet absorbance of attached cells (OD_570 nm_) and the growth of the planktonic cells (OD_600 nm_) to emphasize the amount of cells in a sessile lifestyle at the tested condition. Each point and standard deviation is the mean of 8 samples per condition. Temp, temperature.

To enable rapid quantification of surface-attached biomass, we adapted the commonly used microtitre plate assay based on crystal violet binding [23] for use at high temperatures. To prevent evaporation of the medium it was essential to cover the plates with gas permeable sealing membranes. For incubation at 76°C the plates were placed into a metal container to further prevent evaporation of the medium. The requirements for adherence to abiotic surfaces can vary greatly among microorganisms, therefore, different plates with hydrophilic and hydrophobic surfaces were tested. All three strains, *S. acidocaldarius*, *S. solfataricus* and *S. tokodaii*, attached preferentially to hydrophilic surfaces at the well's walls (data not shown). The conditions for biofilm formation were further optimized for each of the strains. The amount of biomass detected after two days was strongly dependent on the starting OD_600_ of the inoculum and differed for each strain (Fig. S1). Based on these results, for all subsequent experiments the starting OD for *S. acidocaldarius* was 0.01, for *S. solfataricus* was 0.03 and for *S. solfataricus* was 0.06. It was confirmed that the crystal violet values reflected the amount of biomass formed, as the obtained values correlated with the OD values measured from resuspended biofilm cells (data not shown). For the presentation of the microtitre plate assay results we show the correlation of the crystal violet release of the biofilm cells (OD_570 nm_) divided by the growth of the planktonic cells (OD_600 nm_) to emphasize the fraction of the cells which grow as biofilm under each condition in Fig. 1 and the absolute amount of surface-associated cells in Fig. S2.

### Influence of pH, temperature, and iron concentration on *Sulfolobus* biofilm formation

Using the adapted microtitre plate assay the influence of a variety of conditions on the biofilm formation of the three *Sulfolobus* strains was tested. The pH values of a hot spring may be subject to change, for example by incoming rain or changes in the pH of fluid entering the hot spring. Therefore, the effect of pH values ranging from 2 to 7 on biofilm formation were evaluated. As expected, growth of all three strains was optimal around pH 3–4, but at pH 6 up to 80 and 70% of the total biomass of *S. acidocaldarius* and *S. tokodaii*, respectively, was present in biofilm (Fig. 1, second column and Fig. S2, C). Based on the correlation between OD values and the amount of surface-attached biomass, it was evident that in both species biofilm formation protects cells against alkaline pH, as the optimum pH for biofilm formation was much higher than the growth optimum.

As the temperature in a hot spring may also be subject to rapid changes, biofilm formation in the microtitre plates were tested at temperatures ranging from 60–85°C. In the range from 65 to 80°C *S. tokodaii* formed equal amounts of biofilm, with decreased levels only observed at 60 and 85°C, although at 60°C the amount of cells present in biofilms was the highest (50%)(Fig. 1, first column and Fig. S2,B). In contrast, *S. acidocaldarius* and *S. solfataricus* displayed increased biofilm formation at both extremes of the temperature gradient; at 60°C *S. acidocaldarius* and *S. solfataricus* showed a 5-fold and 4-fold increase biofilm formation, respectively, when compared with the optimal growth temperature of 75°C.

The natural habitats of *Sulfolobales* are acidic geothermal springs which are rich in As, S and Fe [24], [25]. In these springs hydrous ferric oxide (HFO) microbial mats are found which contain a variety of members of the *Sulfolobales* indicating that these microorganisms might play a role in mediating the oxidation of iron in these environments [24]. Therefore, the influence of iron concentration on biofilm formation was tested.

Whereas the normal iron concentration in the medium of *Sulfolobales* is 0.02 g/L, we tested 0.015 g/L to 0.065 g/L. Biofilm formation by *S. acidocaldarius* and *S. tokodaii* was not significantly influenced by the different concentrations of iron, but *S. solfataricus* displayed an optimum curve with the highest biofilm formation at 0.045 g/L (Fig. 1, third column and Fig. S2, D). When different pH values and iron conditions were combined, it was interesting to see that *S. solfataricus* was unable to resist the higher pH in the presence of high iron concentrations and, subsequently, biofilm formation was abolished. In contrast *S. tokodaii* and *S. acidocaldarius* biofilm formation was additionally stimulated (Fig. 1, last column and Fig. S2, E). At pH 6 and 0.065 g/L iron, biofilm formation increased 4-fold for *S. tokodaii* and 10-fold for *S. acidocaldarius* which compared with normal levels reaching 63 and 83% of cells, respectively, in biofilm compared to the total cell mass (Table S1 and Fig. S2, E).

In general, the amount of formed biofilm in microtitre plates is much less for *S. solfataricus* than for the other two species, most probably due to the more anaerobic conditions as compared to the static biofilm assay in Petri dishes.

### Structural determination of static biofilms of the three *Sulfolobus* strains by confocal laser scanning electron microscopy

**Figure 2:**
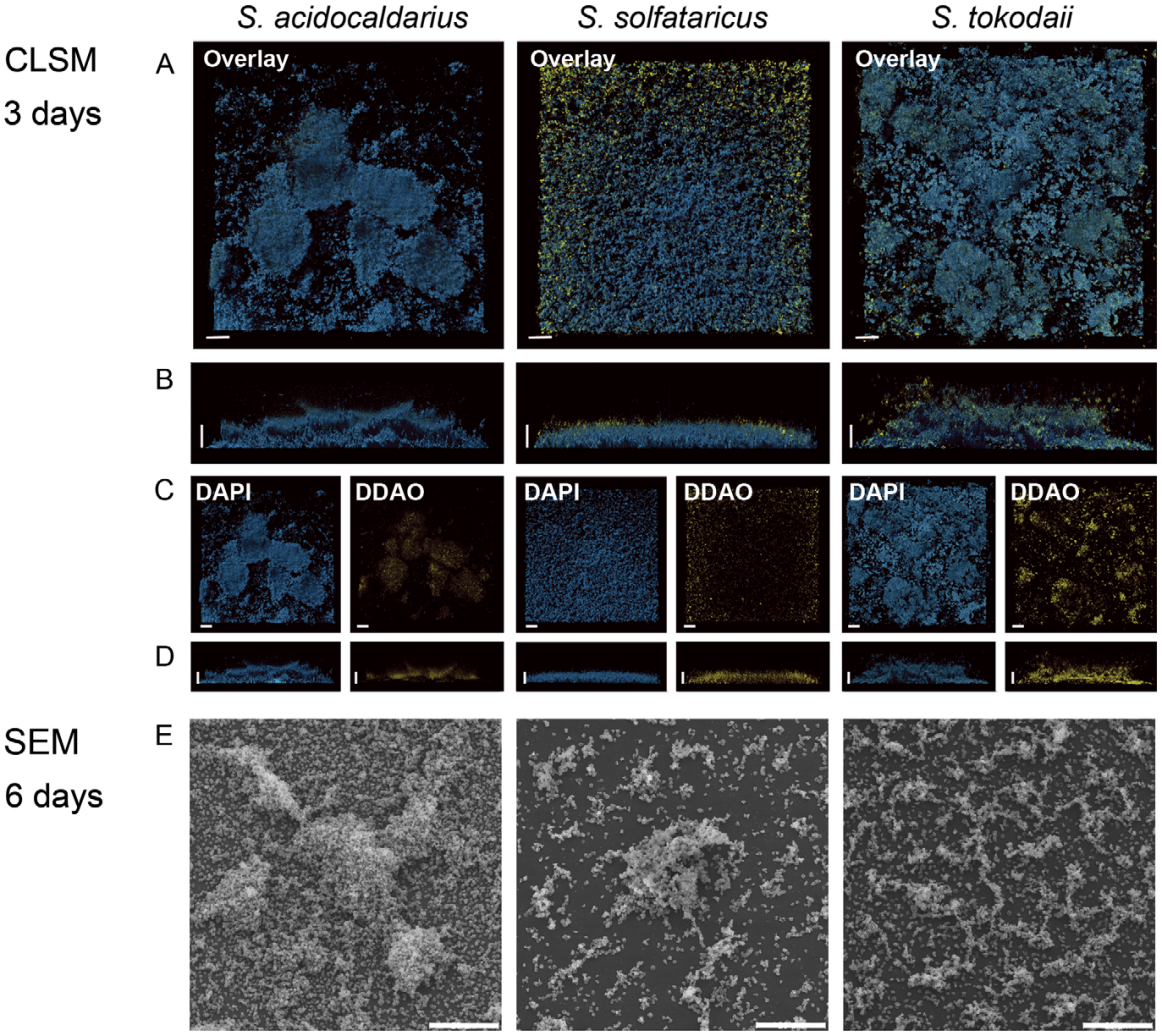
Different structures of static biofilms formed by three *Sulfolobus* strains *S. acidocaldarius*, *S. solfataricus* and *S. tokodaii* visualized by CLSM and SEM. A (top views) and B (side views) display the overlays of the images of three day old biofilms treated with DAPI and DDAO. The bar is 20 µm in length. C (top view) and D (side views) show the single channels of the overlays. DAPI signal: blue; DDAO signal: yellow. E, SEM images of biofilms of the three *Sulfolobus* strains incubated for 6 days. CLSM: confocal laser scanning microscopy; SEM: scanning electron microscopy.

**Figure 3:**
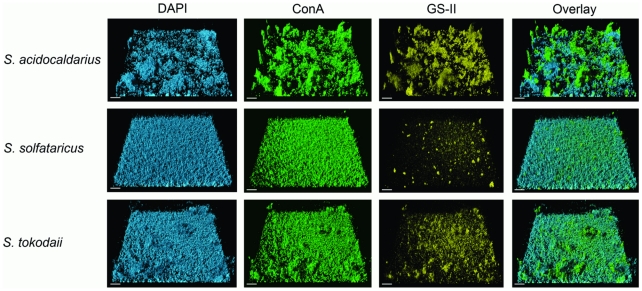
CSLM analysis of three day old static biofilms of *S. acidocaldarius*, *S. solfataricus* and *S. tokodaii* by lectin-staining. After three days the biofilms of *S. acidocaldarius* (first row), *S. solfataricus* (second row) and *S. tokodaii* (last row) were incubated with DAPI and different lectins and were analyzed by CSLM. The first column displays the DAPI signal (blue), the second column the ConA signal (green), the third column the GSII signal (yellow) and the last column the overlay of the other three channels. Bars are 20 µm in length.

All three *Sulfolobus* strains were inoculated in uncoated plastic µ-dishes and incubated without agitation at 76°C. Evaporation was prevented by placing the Petri dishes in a humidified metal box and the medium was carefully exchanged every 24 hrs with fresh, prewarmed medium to ensure nutrient and oxygen availability. After three days the formed biofilms were stained with DAPI, as described in the Materials and Methods section and analyzed by confocal laser scanning microscopy (CLSM). We employed DAPI staining to visualize cells as there is currently no fluorescent protein available that is stably expressed under the growth conditions of *Sulfolobus spp*.. *S. solfataricus* formed biofilms (20–30 µm thick) with a carpet like structure covering the whole surface of the Petri dish with a low density of cells (Fig. 2, middle column). The biofilm structure of *S. tokodaii* was 25–35 µm thick and also exhibited a carpet like structure but, in contrast to *S. solfataricus*, these had a high cell density and, occasionally, cell aggregates were visible (Fig. 3, overlay picture, last row). *S. acidocaldarius* readily formed biofilms (25–35 µm thick) which contained a high density of cells and large aggregates, forming towering structures above the surface of attached cells (Fig. 3, first row).

For bacteria it is well known that extracellular DNA can play an essential role in the formation and stabilization of the three-dimensional structure of biofilms [26]. To examine whether extracellular DNA was present in biofilms of the three *Sulfolobus* strains, three days old biofilms were stained concomitantly with DAPI and the membrane-impermeable DNA-binding dye DDAO. In all three strains the DDAO signal was only present at locations in the biofilm where aggregates were also visible (Fig. 2, middle panels C and D). The weak DDAO signal was further reduced following DNase treatment indicating that the extracellular DNA was removed, but had no effect on the structure of the biofilms. Therefore, at this stage of the biofilm maturation, extracellular DNA does not appear to play a structural role in biofilms of these three *Sulfolobus* strains.

To estimate the amount of living and dead cells three days old biofilms were stained with the LIVE/DEAD stain. In *S. solfataricus* and *S. tokodaii* it was evident that throughout the biofilm less then ∼2% of the cells were dead whereas in *S. acidocaldarius* up to ∼8% of cells were dead (data not shown).

### Analysis of *Sulfolobus* matrix components

The extracellular matrix that connects the cells and enables three-dimensional structuring of the communities is thought to be composed of (glyco)proteins, lipids, extrallular DNA (eDNA), and polymeric carbohydrates [27]. We therefore tested, whether eDNA, proteins, and polysaccharides play an important structural role in *Sulfolobus* biofilms. Experiments to inhibit biofilm formation by the addition of Proteinase K or DNase did not give conclusive results. Irrespective of the time at which DNase or Proteinase K was added, no effect on biofilm formation was observed although both enzymes showed enzyme activity under the conditions tested (data not shown).

Recently, it has been described that *S. solfataricus* cells, particularly when surface-attached, produce extracellular polymeric substances (EPS) containing glucose, mannose, galactose and N-acetyl-glucosamine [18], [28]. To test whether cells also produce EPS during biofilm formation, three days maturated biofilms of all three *Sulfolobus* species were stained with DAPI, and two different fluorescently labeled lectins (Fig. 3). The lectins selected were concanavalin A (ConA), specific for terminal glucose and mannose residues and either IB4 (specific for galactosyl residues) or GSII (specific for N-acetylglucosamine residues). In all three strains it was observed that the ConA signal (green signal) corresponded to the DAPI signal (blue signal, Fig. 3). *Sulfolobus* cells are covered by an S-layer and is has been described that the S-layer proteins are glycosylated and can be purified by ConA affinity chromatography [29], [30], [31]. Whereas in *S. solfataricus* the ConA-derived signal did, in fact, completely overlap with the DAPI signal, in *S. tokodaii* and *S. acidocaldari*us GSII and IB4 lectin (yellow channel) stained matter was observed on top of the cell layer and, as no DAPI signal was found in this accumulated material, we concluded that these two strains secrete EPS. In both strains, these clouds of EPS also reacted with the other two lectins indicating the presence of not only mannose and glucose, but also galactose and N-acetylglucosamine (Fig. S3). In *S. solfataricus*, only marginal GSII- and IB_4_-mediated staining of cell attached sugar residues was observed, indicating a different glycosylation of extracellular proteins than in the other two strains.

**Figure 4:**
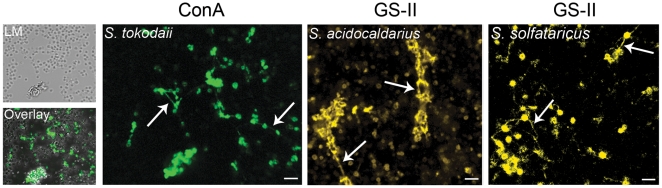
Connections between cells in three days matured static biofilms of *S. acidocaldarius*, *S. tokodaii* and *S. solfataricus.* The left three pictures show the CLSM analysis of a ConA treated *S. tokodaii* biofilm (LM: light microscopy picture, ConA: green channel, Overlay: overlay of the ConA signal and the LM picture). Middel panel: CLSM analysis of GS-II (yellow) treated *S. acidocaldarius* biofilm. Right panel: CLSM analysis of GS-II (yellow) treated *S. solfataricus* biofilm. Arrows indicate the connections. Bars are 4,5 µm in length. CLSM: confocal laser scanning microscopy; LC: light microscopy.

**Figure 5:**
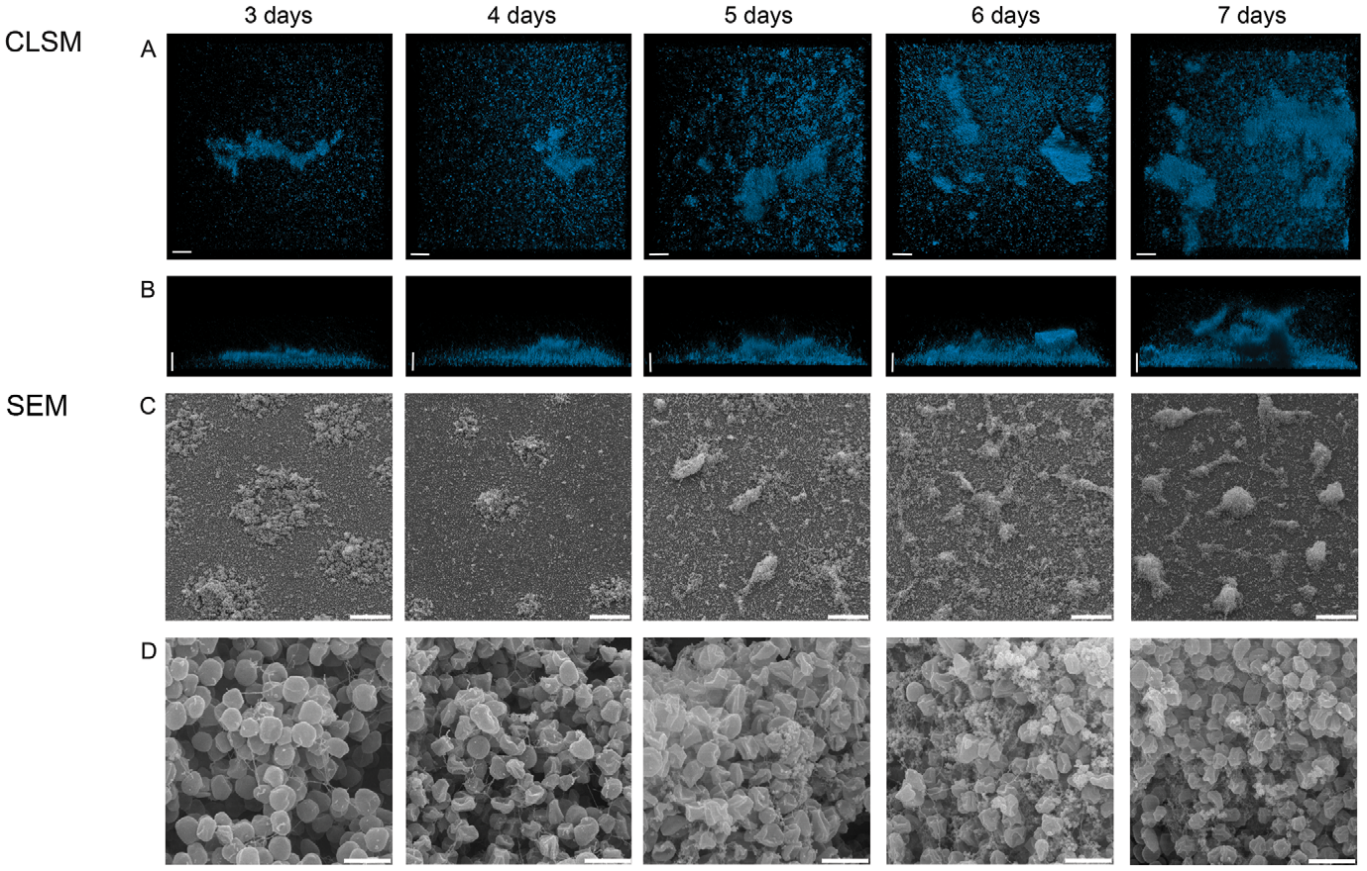
CLSM and SEM analysis of the development of a static biofilm of *S. acidocaldarius* during a time course of seven days. DAPI signal (blue) in the top view (A) and the side view (B). SEM analysis showing an overview (C) and enlarged view (D) of the developing biofilm. Size standards are 20 µM in length for A and B, 40 µM in C and 2 µM in D. CLSM: confocal laser scanning microscopy; SEM: scanning electron microscopy.

A detailed analysis of a biofilm formed by *S. tokodaii* and *S. acidocaldarius* showed extensive cell-cell connections. These connections became visible when the *S. tokodaii* sample was incubated with ConA and analysed by CLSM (Fig. 4). The connection might be a string of sugars or flagella/pili in which the subunits are glycosylated. In *S. acidocaldarius* and *S. solfataricus* biofilms the lectin GSII also stained thin connections between the cells (Fig. 4) which were clearly visible also in SEM pictures (Fig. 5D).

### Maturation of *S. acidocaldarius* over a range of seven days

**Figure 6:**
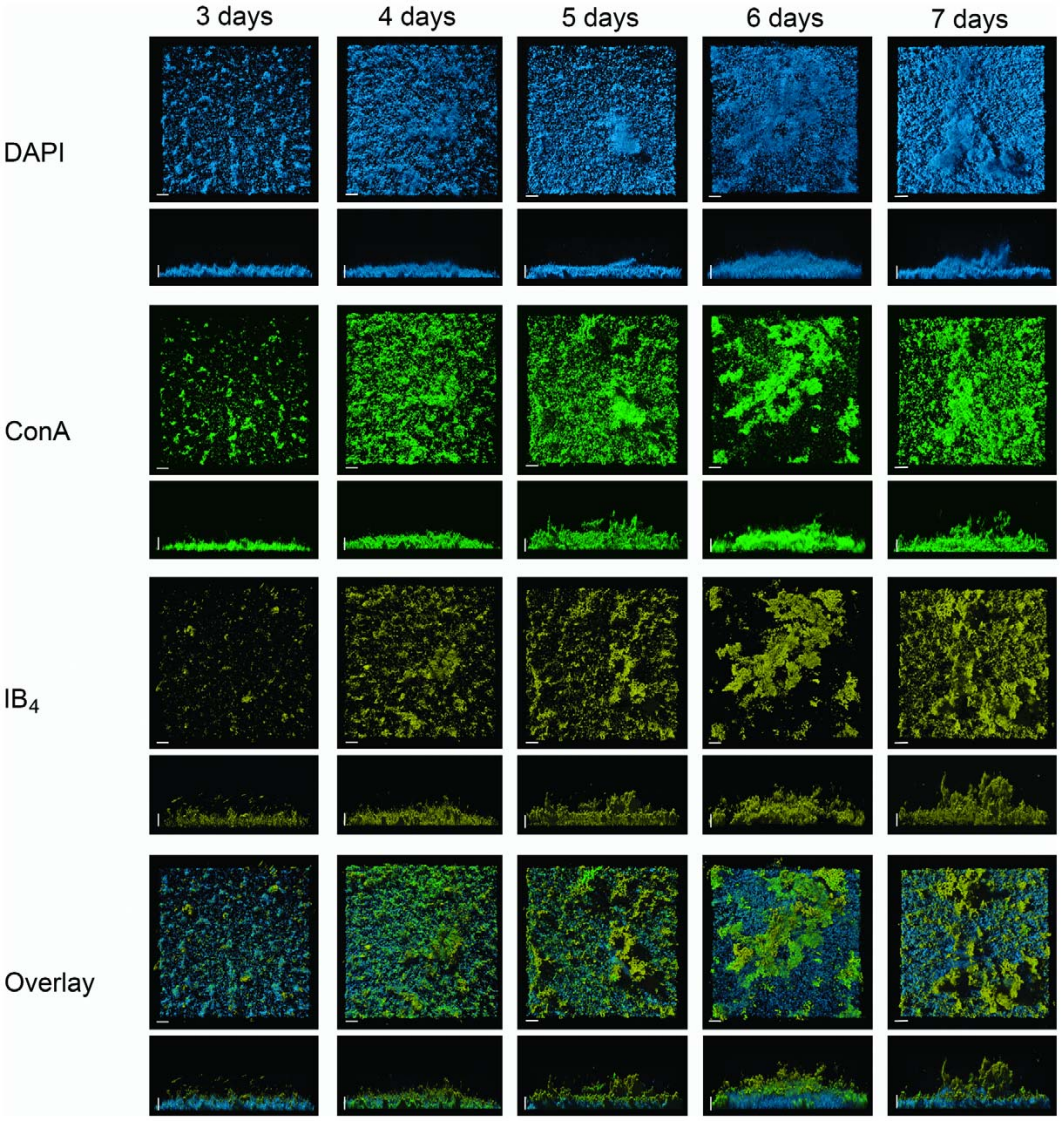
Lectin-based analysis of developing static biofilm of *S. acidocaldarius* during a time course of seven days. Samples were treated with DAPI (blue channel), Con A (green channel) and IB_4_ (yellow channel) and analyzed by CSLM. For each channel the top view and the side view is presented. An overlay shows all three channels. Bars are 20 µm in length. CLSM: confocal laser scanning microscopy.

All experiments described so far were performed using 2–3 days old biofilms of *Sulfolobus* spp.. In order to monitor further community development under static conditions, biofilms of *S. acidocaldarius* were allowed to develop for seven days. Each day one sample was treated with DAPI and analyzed by CLSM. The thickness of the biofilm increased from 30 µM in height on day three to 150 µM (including EPS structures) on day seven (Fig. 5AB). For a more detailed analysis of the maturation of biofilm formation by *S. acidocaldarius* the cells were inoculated in large Petri dishes in which polylysine covered glass slides had been placed. These slides were then analyzed by scanning electron microscopy (SEM). Only 15 minutes after the addition of the cell suspension a few cells attached to the surface, and some budding of vesicles was visible (Fig. S4A). After two hours there was not an apparent increase in the number of attached cells, but nearly all attached cells had formed filamentous structures adhering the cells to the surface or neighboring cells (Fig. S4B). After 36 hours, microcolonies started to form with only a few cells remaining on the rest of the surface whereas after 48 hours the surface of the glass plates was completely covered with cells. In the microcolonies, cells appeared to be connected by a network of filamentous structures as was observed previously following lectin staining (Fig. 5D). These connections grew denser and also increasing extracellular material accumulated in the later stages of the biofilm formation (Fig. 5D). Interestingly, on the seventh day the layer of cells at the surface of the glass slide apparently disappeared and the density of cells in the detailed view was reduced compared with the sixth day (Fig. 5C/D). To test whether the extracellular material visible in the closer SEM view of the towering structures did indeed consist of EPS, *S. acidocaldarius* biofilms were incubated for seven days, stained with lectins, as described above, and analyzed by CLSM (Fig. 6). Towering structures were formed which were initiated by the secretion of EPS and then colonized by cells.

The secretion of certain sugars apparently progressed in a sequential manner: initially the ConA (glucose and mannose) derived signal was much stronger, but in later stages both the GSII (galactose) and the IB4 (N-acetylgucosamine) signal increased (Fig. 6 and Fig. S5), after which the secretion of mannose-rich sugars increases again as detected by ConA. This indicates that these sugars play an important role in biofilm maturation.

### Role of surface appendages on static biofilm formation in *S. solfataricus*

Attachment of *S. solfataricus* cells from shaking cultures to different surfaces is mediated by flagella and UV induced pili. Deletion mutants in which either the *flaJ* gene, encoding the integral membrane protein of the flagella operon, or the *uspE* gene, encoding the ATPase of the UV-induced pili operon were incapable of attachment to glass surfaces, gold-coated carbon grids or mica [18]. In bacterial biofilm development, filaments such as type IV pili and flagella have a strong effect on the dynamics of biofilm formation. Therefore, we tested the Δ*flaJ* and Δ*upsE* mutants and the wild type *S. solfataricus* PBL2025 strain for their ability to form static biofilms in three days. PBL2025 is a *S. solfataricus* strain which lacks 50 genes predicted to be involved in sugar metabolism and uptake and is the only currently available *S. solfataricus* strain which can be genetically manipulated [22].

**Figure 7:**
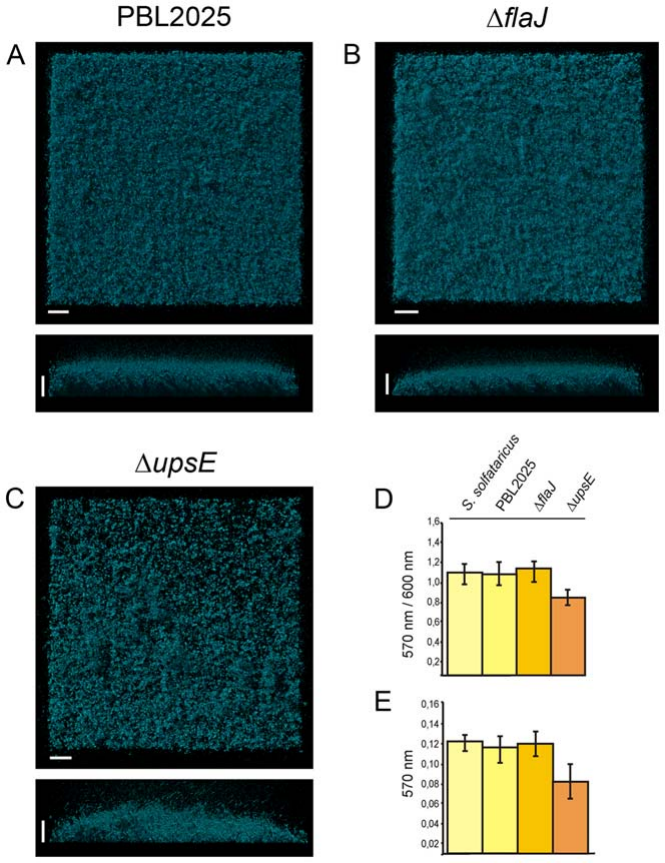
Three day matured static biofilms of S. solfataricus PBL2025, Δ*flaJ* and Δ*upsE*. Biofilms of PBL2025, Δ*flaJ* and Δ*upsE* were stained with DAPI and analyzed by CLSM (A–C, respectively). Complementary, a microtitre plate assay was performed for 72 hrs with all three strains and biofilm formation is presented in D as the correlation of the crystal violet absorbance (OD_570 nm_) divided by the optical density of the planktonic cells (OD_600 nm_) and in E the crystal violet absorbance (OD_570 nm_) is indicated. Bars are 20 µm in lengt.

The PBL2025, Δ*flaJ* and Δ*upsE* strains were grown in petri dishes and tested in the microtitre plate assay. After three days the matured biofilms in the petri dishes were stained with DAPI and analyzed by CLSM. The biofilms of PBL2025 and the Δ*flaJ strain* were comparable to three day old biofilms of *S. solfataricus* in height, density and structure, and showed mainly a carpet like appearance (Fig. 7A and B). However, in biofilms from the Δ*upsE strain*, the surface of the petri dish was not as completely covered with cells and the biofilm was less dense as compared to PBL2025 and the Δ*flaJ* mutant strain. Furthermore, slightly more aggregates were visible in the biofilms of the Δ*upsE* strain. These observations were supported by the results of the microtitre plate assay showing that only the Δ*upsE* strain produced significantly less biofilm than *S. solfataricus*, PBL2025 and Δ*flaJ*. We therefore concluded that cell appendages do not to play an important role in the early development of static biofilm formation in *S. solfataricus* strains.

## Discussion

It is well known that archaea and bacteria coexist in natural biofilms, playing essential roles in the Earth's biogeochemical cycles as well as in human disease [2]. The formation of bacterial biofilms has been very well documented. Studies have been carried out on euryarchaeal biofilm formation whereas we presented here the first detailed insights into crenarchaeal biofilm formation.

We chose the thermoacidophilic crenarchaeotes *Sulfolobus* spp as a model to establish methods for the analysis of hyperthermophilic archaeal biofilm formation. *Sulfolobus* spp. exist in acidic, mostly muddy, hot springs all over the world in which the hydrological dynamics result in rapid variations in temperature, pH and geochemical conditions. Therefore, these organisms must quickly adapt to these changing conditions or exist in a state that enables them remain undisturbed by such changes. As fully maturated biofilms protect their inhabitants from environmental disturbances, this form might be a way for *Sulfolobus* spp to survive in their habitats. The three selected strains were originally isolated from well separated geographical locations and each of the strains did, in fact, behave differently following the initiation of biofilm formation. Of the three strains, *S. acidocaldarius* most readily engaged in community formation either in microtitre plate assays or in static biofilm conditions when compared with the other *Sulfolobus* strains. In particular, when challenged with low temperature (60°C) or the combination of near neutral pH and low iron concentrations, *S. acidocaldarius* responded with highly increased biofilm formation demonstrating the ability of this strain to adapt to rapid changes in temperature and pH.

The maturation of bacterial biofilms proceeds via defined steps including initial attachment and further development into microcolonies secreting extrapolymeric substances [32]. During maturation multilayered biofilm structures are shaped and kept together by the secretion of EPS, extracellular DNA, and proteins [27], [33]. Cells can be released from matured biofilms at any time point to proceed with a planktonic life style. Very recently, we have shown that *S. solfataricus* displays flagella and pili-dependent attachment to abiotic surfaces [18]. After two days of attachment to a glass slide in a shaking culture the cells started to produce EPS which contained glucose, mannose, galactose and N-acetylglucosamine demonstrating the first phase of biofilm formation. Similar to bacterial biofilm formation, it is evident that after initial attachment *Sulfolobus* cells start to form microcolonies that are surrounded by an extracellular matrix, containing EPS and, most probably, proteins. The function of EPS formation in these *Sulfolobus* strains may serve a variety of purposes. A natural deletion mutant of *S. solfataricus* which lacks 50 genes overproduced EPS when attached to a glass slide [18]. The deleted region contains genes mainly involved in sugar degradation and transport and these were shown to be upregulated in attached *S. solfataricus* cells, implying that they play an important role in the modulation of the EPS. One might speculate that the EPS is used as a carbohydrate reservoir which might also be the case when the cells are engaged in biofilm formation. When the *S. acidocaldarius* biofilm was incubated for seven days it was evident that the different sugars were produced in a consecutive manner implying that they may serve different purposes. Moreover, a layer of EPS was produced which enabled the formation of three-dimensional tower-like structures, especially in *S. acidocaldarius*. It appeared that after seven days the *S. acidocaldarius* biofilms detached by releasing attached cells to the planktonic phase.

In bacteria, cell appendages such as type IV pili and flagella are very well known for their influence and importance in the dynamics and development of static and hydrodynamically grown biofilms [34]. Like in *Vibrio cholerae* and *Shewanella oneidensis* MR-1, the *S. solfataricus* pili mutant Δ*upsE* exhibited decreased biofilm formation in the microtitre plate assay [35], [36]. Also more dense aggregates were observed as in *Pseudomonas aeruginoasa* and *S. oneidensis* MR-1 type IV pili mutants [36], [37]. However, the flagella mutant showed no obvious differences in static biofilm formation to the wild type, As the *S. solfataricus* flagella and pili mutant could not attach to several different surfaces in shaking culture [18], it will be interesting for future studies whether flagella and pili have a greater impact on biofilm formation in flow chamber systems.

Taken together, we demonstrated that *Sulfolobus* species can engage in biofilm formation and developed methods to study these in more detail. Of the three strains, *S. acidocaldarius* formed the largest amounts of biomass and was able to evade unfavorable conditions most successfully by choosing this life style. Interestingly, these data support the observation that *S. acidocaldarius* is mainly sampled from the crusts surrounding acidic hot springs and mud holes (Karl-Otto Stetter, personal communication) whereas *S. solfataricus* and *S. tokodaii* are primarily isolated from the midst of these types of hot springs, where the hot fluids are bubbling up to the surface [38] (Christa Schleper, personal communication).

In the future, it will be interesting to study how *Sulfolobus* strains behave in mixed biofilms and even in communities including other inhabitants of these acidic hot springs.

## Supporting Information

Table S1 (0.12 MB DOC)

Figure S1 Optimization of inoculation conditions for biofilm formation of S. acidocaldarius, S. solfataricus and S. tokodaii. The strains were inoculated with different OD 600 and incubated in a microtitre plate for three days. The correlation of the measured crystal violet absorbance of the formed biofilm and the OD600 values of the planktonic cells is presented. Each bar represents the mean of 8 different samples.
(0.23 MB TIF)

Figure S2 Data shown in Figure 1 presented as calculated percentage of cells within the biofilm related to the total amount of cells in biofilm and planktonic cells. (A) Biofilms were grown and either resuspended by prolonged vortexing to obtain the OD600nm, or stained with crystal violet to obtain the OD570nm values. This relation was used to calculate the percentage of cells within the biofilm related to the total amount of cells in biofilm and planktonic cells for (B) different temperatures, (C), different pH values, (D) different iron concentrations, and (E) a combination of different iron concentrations and pH values (D). S. acidocaldarius (blue), S. tokodaii (green) and S. solfataricus (red) are indicated by different colors.
(0.49 MB TIF)

Figure S3 CSLM analysis of three day old static biofilms of S. acidocaldarius, S. solfataricus and S. tokodaii by lectins. After three days the biofilms of S. acidocaldarius (first row), S. solfataricus (second row) and S. tokodaii (last row) were incubated with DAPI and different lectins and were analyzed by CSLM. The first column shows the DAPI signal (blue), the second column the Con A signal (green), the third column the IB4 signal (yellow) and the last column the overlay of the other three channels. Bars are 20 μm in length. CLSM: confocal laser scanning microscopy.
(5.80 MB TIF)

Figure S4 SEM pictures from early stages of S. acidocaldarius biofilm formation from 15 minutes to 48 hours after incubation. (A) shows the overviews and (B) and (C) more detailed views of the respective picture in A in the same column of the developing biofilms. The length of the bars is indicated in the images. SEM: scanning electron microscopy.
(7.76 MB TIF)

Figure S5 Lectin based analysis of developing static biofilm of S. acidocaldarius. Samples were treated with DAPI (blue channel), Con A (green channel) and GSII (yellow channel) and analyzed by CSLM. For each channel the top view and the side view is presented. Overlay shows all three channels again including top- and side views. Bars are 20 μm in length.
(9.90 MB TIF)

## References

[1] Costerton JW, Lewandowski Z, Caldwell DE, Korber DR, Lappin-Scott HM (1995). Microbial biofilms.. Annu Rev Microbiol.

[2] Hall-Stoodley L, Costerton JW, Stoodley P (2004). Bacterial biofilms: from the natural environment to infectious diseases.. Nat Rev Microbiol.

[3] Moons P, Michiels CW, Aertsen A (2009). Bacterial interactions in biofilms.. Crit Rev Microbiol.

[4] Hall-Stoodley L, Stoodley P (2005). Biofilm formation and dispersal and the transmission of human pathogens.. Trends Microbiol.

[5] Parsek MR, Singh PK (2003). Bacterial biofilms: an emerging link to disease pathogenesis.. Annu Rev of Microbiol.

[6] Kruger M, Blumenberg M, Kasten S, Wieland A, Kanel L (2008). A novel, multi-layered methanotrophic microbial mat system growing on the sediment of the Black Sea.. Environ Microbiol.

[7] Zhang CL, Ye Q, Huang Z, Li W, Chen J (2008). Global occurrence of archaeal *amo*A genes in terrestrial hot springs.. Appl Environ Microbiol.

[8] Rinker KD, Kelly RM (1996). Growth physiology of the hyperthermophilic archaeon *Thermococcus litoralis*: Development of a sulfur-free defined medium, characterization of an exopolysaccharide, and evidence of biofilm formation.. Appl Environ Microbiol.

[9] Lapaglia C, Hartzell PL (1997). Stress-induced production of biofilm in the hyperthermophile *Archaeoglobus fulgidus*.. Appl Environ Microbiol.

[10] Näther DJ, Rachel R, Wanner G, Wirth R (2006). Flagella of *Pyrococcus furiosus*: multifunctional organelles, made for swimming, adhesion to various surfaces, and cell-cell contacts.. J Bacteriol.

[11] Thoma C, Frank M, Rachel R, Schmid S, Nather D (2008). The Mth60 fimbriae of *Methanothermobacter thermoautotrophicus* are functional adhesins.. Environ Microbiol.

[12] Schopf S, Wanner G, Rachel R, Wirth R (2008). An archaeal bi-species biofilm formed by *Pyrococcus furiosus* and *Methanopyrus kandleri*.. Arch Microbiol.

[13] Baker-Austin C, Potrykus J, Wexler M, Bond PL, Dopson M (2010). Biofilm development in the extremely acidophilic archaeon ‘*Ferroplasma acidarmanus*’ Fer1.. Extremophiles.

[14] Albers SV, Pohlschroder M (2009). Diversity of archaeal type IV pilin-like structures.. Extremophiles.

[15] Fröls S, Ajon M, Wagner M, Teichmann D, Zolghadr B (2008). UV-inducible cellular aggregation of the hyperthermophilic archaeon *Sulfolobus solfataricus* is mediated by pili formation.. Mol Microbiol.

[16] Szabo Z, Sani M, Groeneveld M, Zolghadr B, Schelert J (2007). Flagellar motility and structure in the hyperthermoacidophilic archaeon *Sulfolobus solfataricus*.. J Bacteriol.

[17] Fröls S, Ajon M, Wagner M, Teichmann D, Zolghadr B (2008). UV-inducible cellular aggregation of the hyperthermophilic archaeon *Sulfolobus solfataricus* is mediated by pili formation.. Mol Microbiol.

[18] Zolghadr B, Klingl A, Koerdt A, Driessen AJM, Rachel R (2010). Appendage-mediated surface adherence of *Sulfolobus solfataricus*.. J Bacteriol.

[19] Zillig W, Stetter KO, Wunderl S, Schulz W, Priess H (1980). The Sulfolobus – “ Caldariella ” Group: Taxonomy on the basis of the structure of DNA-dependent RNA polymerases.. Arch Microbiol.

[20] Brock TD, Brock KM, Belly RT, Weiss RL (1972). *Sulfolobus*: a new genus of sulfur-oxidizing bacteria living at low pH and high temperature.. Arch Microbiol.

[21] Suzuki T, Iwasaki T, Uzawa T, Hara K, Nemoto N (2002). *Sulfolobus tokodaii* sp. nov. (f. *Sulfolobus* sp. strain 7), a new member of the genus *Sulfolobus* isolated from Beppu Hot Springs, Japan.. Extremophiles.

[22] Schelert J, Dixit V, Hoang V, Simbahan J, Drozda M (2004). Occurrence and characterization of mercury resistance in the hyperthermophilic archaeon *Sulfolobus solfataricus* by use of gene disruption.. J Bacteriol.

[23] O'Toole GA, Kolter R (1998). Initiation of biofilm formation in *Pseudomonas fluorescens* WCS365 proceeds via multiple, convergent signalling pathways: a genetic analysis.. Mol Microbiol.

[24] Macur RE, Langner HW, Kocar BD, Inskeep WP (2004). Linking geochemical processes with microbial community analysis: successional dynamics in an arsenic-rich, acid-sulfate-cloride geothermal spring.. Geobiology.

[25] Inskeep WP, Macur RE, Harrison G, Bostick BC, Fendorf S (2004). Biomineralization of As(V)-hydrous ferric oxyhydroxide in microbial mats of an acid-sulfate-chloride geothermal spring, Yellowstone National Park.. Geochim Cosmochim Acta.

[26] Allesen-Holm M, Barken KB, Yang L, Klausen M, Webb JS (2006). A characterization of DNA release in *Pseudomonas aeruginosa* cultures and biofilms.. Mol Microbiol.

[27] Flemming HC, Neu TR, Wozniak DJ (2007). The EPS matrix: the “house of biofilm cells”.. J Bacteriol.

[28] Nicolaus B, Manca MC, Romano I, Lama L (2003). Production of an exopolysaccharide from two thermophilic archaea belonging to the genus *Sulfolobus*.. FEMS Microbiol Lett.

[29] Grogan DW (1996). Isolation and fractionation of cell envelope from the extreme thermo-acidophile *Sulfolobus acidocaldarius.*. J Microbiol Meth.

[30] Veith A, Klingl A, Zolghadr B, Lauber K, Mentele R (2009). *Acidianus*, *Sulfolobus* and *Metallosphaera* surface layers: structure, composition and gene expression.. Mol Microbiol.

[31] Ellen AF, Albers SV, Huibers W, Pitcher A, Hobel CF (2009). Proteomic analysis of secreted membrane vesicles of archaeal *Sulfolobus* species reveals the presence of endosome sorting complex components.. Extremophiles.

[32] Sauer K, Camper AK, Ehrlich GD, Costerton JW, Davies DG (2002). *Pseudomonas aeruginosa* displays multiple phenotypes during development as a biofilm.. J Bacteriol.

[33] Sutherland IW (2001). The biofilm matrix—an immobilized but dynamic microbial environment.. Trends Microbiol.

[34] Karatan E, Watnick P (2009). Signals, regulatory networks, and materials that build and break bacterial biofilms.. Microbiol Mol Biol Rev.

[35] Watnick PI, Kolter R (1999). Steps in the development of a *Vibrio cholerae* El Tor biofilm.. Mol Microbiol.

[36] Thormann KM, Saville RM, Shukla S, Pelletier DA, Spormann AM (2004). Initial phases of biofilm formation in *Shewanella oneidensis* MR-1.. J Bacteriol.

[37] Chiang P, Burrows LL (2003). Biofilm formation by hyperpiliated mutants of *Pseudomonas aeruginosa*.. J Bacteriol.

[38] Zillig W, Kletzin A, Schleper C, Holz I, Janekovic D (1994). Screening for *Sulfolobales*, their plasmids and their viruses in Icelandic solfataras.. Syst Appl Microbiol.

