# Supplementary material for: Crenarchaeal Biofilm Formation under Extreme Conditions
Source: PLoS One. 2010 Nov 24;5(11):e14104. doi: 10.1371/journal.pone.0014104 (PMC2991349; doi:10.1371/journal.pone.0014104)
Supplement: Table S1 — (0.12 MB DOC) [file pone.0014104.s001.doc]

**Table S1 Calculation of OD_600_ values from OD_570_ values from two days old biofilms**

| Condition | | | ^P^OD_600_ planktonic cell | crystal violet absorbance  [570 nm] | ^B^OD_600_ biofilm cells [y=5.99x] | Total biomass  [^B^OD_600_+^P^OD_600_] | Cells in biofilm [%] |
| --- | --- | --- | --- | --- | --- | --- | --- |
| Temperature [°C] | |  |  |  |  |  |  |
| *S. acidocaldarius* | | 60 | 0.004 | 0.039 | 0.007 | 0.011 | 61.9 |
|  | | 65 | 0.049 | 0.154 | 0.026 | 0.075 | 34.4 |
|  | | 70 | 0.077 | 0.207 | 0.035 | 0.112 | 31.0 |
|  | | 75 | 0.11 | 0.204 | 0.034 | 0.144 | 23.6 |
|  | | 80 | 0.12 | 0.138 | 0.023 | 0.143 | 16.1 |
|  | | 85 | 0.05 | 0.209 | 0.035 | 0.085 | 41.1 |
| *S.solfataricus* | | 60 | 0.012 | 0.018 | 0.003 | 0.015 | 20.0 |
|  | | 65 | 0.035 | 0.058 | 0.010 | 0.045 | 21.7 |
|  | | 70 | 0.064 | 0.025 | 0.004 | 0.068 | 6.1 |
|  | | 75 | 0.066 | 0.058 | 0.010 | 0.076 | 12.8 |
|  | | 80 | 0.062 | 0.081 | 0.014 | 0.076 | 17.9 |
|  | | 85 | 0.071 | 0.177 | 0.030 | 0.101 | 29.4 |
| *S.tokodaii* | | 60 | 0.01 | 0.055 | 0.009 | 0.019 | 47.9 |
|  | | 65 | 0.03 | 0.067 | 0.011 | 0.041 | 27.2 |
|  | | 70 | 0.054 | 0.103 | 0.017 | 0.071 | 24.2 |
|  | | 75 | 0.06 | 0.141 | 0.024 | 0.084 | 28.2 |
|  | | 80 | 0.061 | 0.139 | 0.023 | 0.084 | 27.6 |
|  | | 85 | 0.096 | 0.167 | 0.028 | 0.124 | 22.5 |
| pH | |  |  |  |  |  |  |
| *S. acidocaldarius* | | 2 | 0.051 | 0.172 | 0.029 | 0.080 | 36.0 |
|  | 3 | | 0.048 | 0.232 | 0.039 | 0.087 | 44.7 |
|  | 4 | | 0.032 | 0.251 | 0.042 | 0.074 | 56.7 |
|  | 5 | | 0.013 | 0.136 | 0.023 | 0.036 | 63.6 |
|  | 6 | | 0.006 | 0.121 | 0.020 | 0.026 | 77.1 |
|  | 7 | | 0.006 | 0.012 | 0.002 | 0.008 | 25.0 |
| *S.solfataricus* | 2 | | 0.042 | 0.019 | 0.003 | 0.045 | 7.0 |
|  | 3 | | 0.041 | 0.137 | 0.023 | 0.064 | 35.8 |
|  | 4 | | 0.045 | 0.049 | 0.008 | 0.053 | 15.4 |
|  | 5 | | 0.038 | 0.077 | 0.013 | 0.051 | 25.3 |
|  | 6 | | 0.035 | 0.097 | 0.016 | 0.051 | 31.6 |
|  | 7 | | 0.019 | 0.051 | 0.009 | 0.028 | 30.9 |
| *S.tokodaii* | 2 | | 0.059 | 0.079 | 0.013 | 0.072 | 18.3 |
|  | 3 | | 0.049 | 0.231 | 0.039 | 0.088 | 44.0 |
|  | 4 | | 0.036 | 0.225 | 0.038 | 0.074 | 51.1 |
|  | 5 | | 0.021 | 0.21 | 0.035 | 0.056 | 62.5 |
|  | 6 | | 0.012 | 0.167 | 0.028 | 0.040 | 69.9 |
|  | 7 | | 0.004 | 0.026 | 0.004 | 0.008 | 52.0 |
| Iron [g/L] |  | |  |  |  |  |  |
| *S. acidocaldarius* | 0.015 | | 0.103 | 0.3 | 0.050 | 0.153 | 32.7 |
|  | 0.025 | | 0.076 | 0.281 | 0.047 | 0.123 | 38.2 |
|  | 0.035 | | 0.078 | 0.286 | 0.048 | 0.126 | 38.0 |
|  | 0.045 | | 0.093 | 0.292 | 0.049 | 0.142 | 34.4 |
|  | 0.055 | | 0.085 | 0.311 | 0.052 | 0.137 | 37.9 |
|  | 0.065 | | 0.07 | 0.313 | 0.052 | 0.122 | 42.7 |
| *S.solfataricus* | 0.015 | | 0.06 | 0.03 | 0.005 | 0.065 | 7.7 |
|  | 0.025 | | 0.045 | 0.036 | 0.006 | 0.051 | 11.8 |
|  | 0.035 | | 0.043 | 0.035 | 0.006 | 0.049 | 12.0 |
|  | 0.045 | | 0.042 | 0.077 | 0.013 | 0.055 | 23.4 |
|  | 0.055 | | 0.037 | 0.051 | 0.009 | 0.046 | 18.7 |
|  | 0.065 | | 0.035 | 0.027 | 0.005 | 0.040 | 11.4 |
| *S.tokodaii* | 0.015 | | 0.068 | 0.096 | 0.016 | 0.084 | 19.1 |
|  | 0.025 | | 0.037 | 0.094 | 0.016 | 0.053 | 29.8 |
|  | 0.035 | | 0.044 | 0.122 | 0.020 | 0.064 | 31.6 |
|  | 0.045 | | 0.036 | 0.12 | 0.020 | 0.056 | 35.8 |
|  | 0.055 | | 0.05 | 0.147 | 0.025 | 0.075 | 32.9 |
|  | 0.065 | | 0.034 | 0.156 | 0.026 | 0.060 | 43.4 |
| pH/Iron [g/L] |  | |  |  |  |  |  |
| *S. acidocaldarius* | 3/0.02 | | 0.148 | 0.165 | 0.028 | 0.176 | 15.7 |
|  | 6/0.045 | | 0.051 | 0.329 | 0.055 | 0.106 | 51.9 |
|  | 6/0.065 | | 0.006 | 0.188 | 0.031 | 0.037 | 84.0 |
| *S.solfataricus* | 3/0.02 | | 0.108 | 0.032 | 0.005 | 0.113 | 4.7 |
|  | 6/0.045 | | 0.051 | 0.008 | 0.001 | 0.052 | 2.6 |
|  | 6/0.065 | | 0.024 | 0.002 | 0.000 | 0.024 | 1.4 |
| *S.tokodaii* | 3/0.02 | | 0.089 | 0.122 | 0.020 | 0.109 | 18.6 |
|  | 6/0.045 | | 0.029 | 0.125 | 0.021 | 0.050 | 41.8 |
|  | 6/0.065 | | 0.007 | 0.071 | 0.012 | 0.019 | 62.9 |
