# Supplementary figures and images for: Crenarchaeal Biofilm Formation under Extreme Conditions
Source: PLoS One. 2010 Nov 24;5(11):e14104. doi: 10.1371/journal.pone.0014104 (PMC2991349; doi:10.1371/journal.pone.0014104)

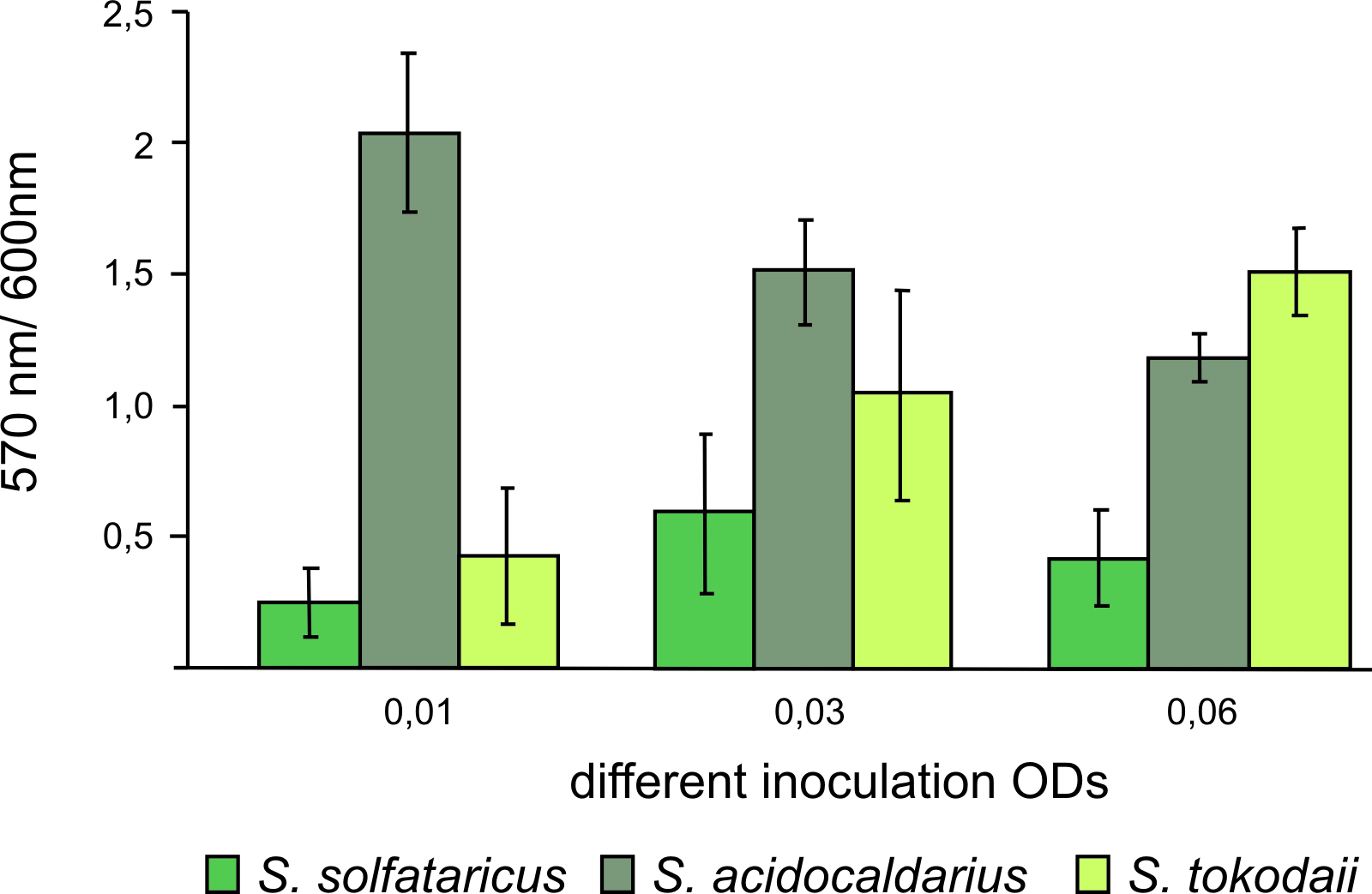

Supplement: Figure S1 — Optimization of inoculation conditions for biofilm formation of S. acidocaldarius, S. solfataricus and S. tokodaii. The strains were inoculated with different OD 600 and incubated in a microtitre plate for three days. The correlation of the measured crystal violet absorbance of the formed biofilm and the OD600 values of the planktonic cells is presented. Each bar represents the mean of 8 different samples. (0.23 MB TIF) [file pone.0014104.s002.tif]

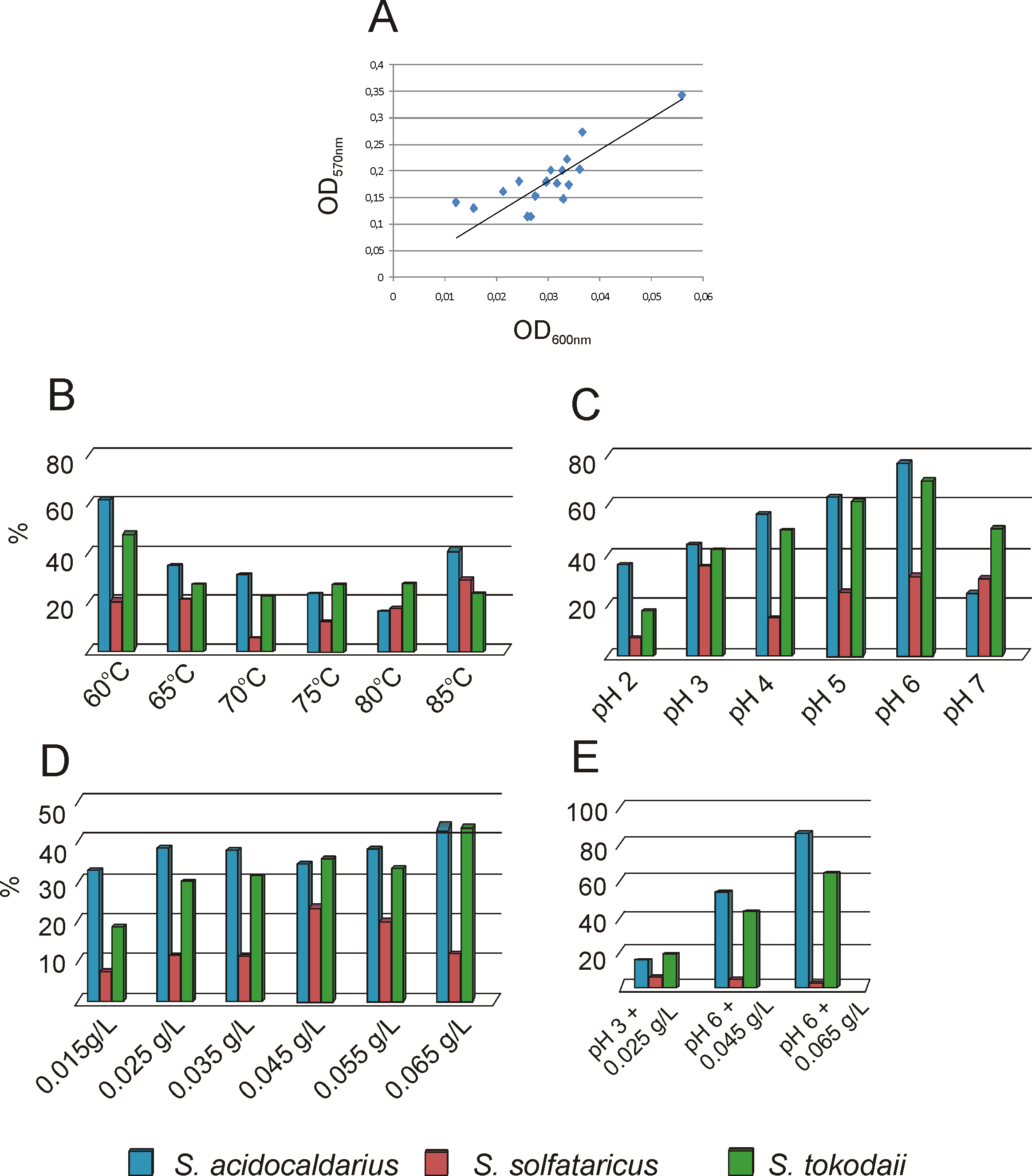

Supplement: Figure S2 — Data shown in Figure 1 presented as calculated percentage of cells within the biofilm related to the total amount of cells in biofilm and planktonic cells. (A) Biofilms were grown and either resuspended by prolonged vortexing to obtain the OD600nm, or stained with crystal violet to obtain the OD570nm values. This relation was used to calculate the percentage of cells within the biofilm related to the total amount of cells in biofilm and planktonic cells for (B) different temperatures, (C), different pH values, (D) different iron concentrations, and (E) a combination of different iron concentrations and pH values (D). S. acidocaldarius (blue), S. tokodaii (green) and S. solfataricus (red) are indicated by different colors. (0.49 MB TIF) [file pone.0014104.s003.tif]

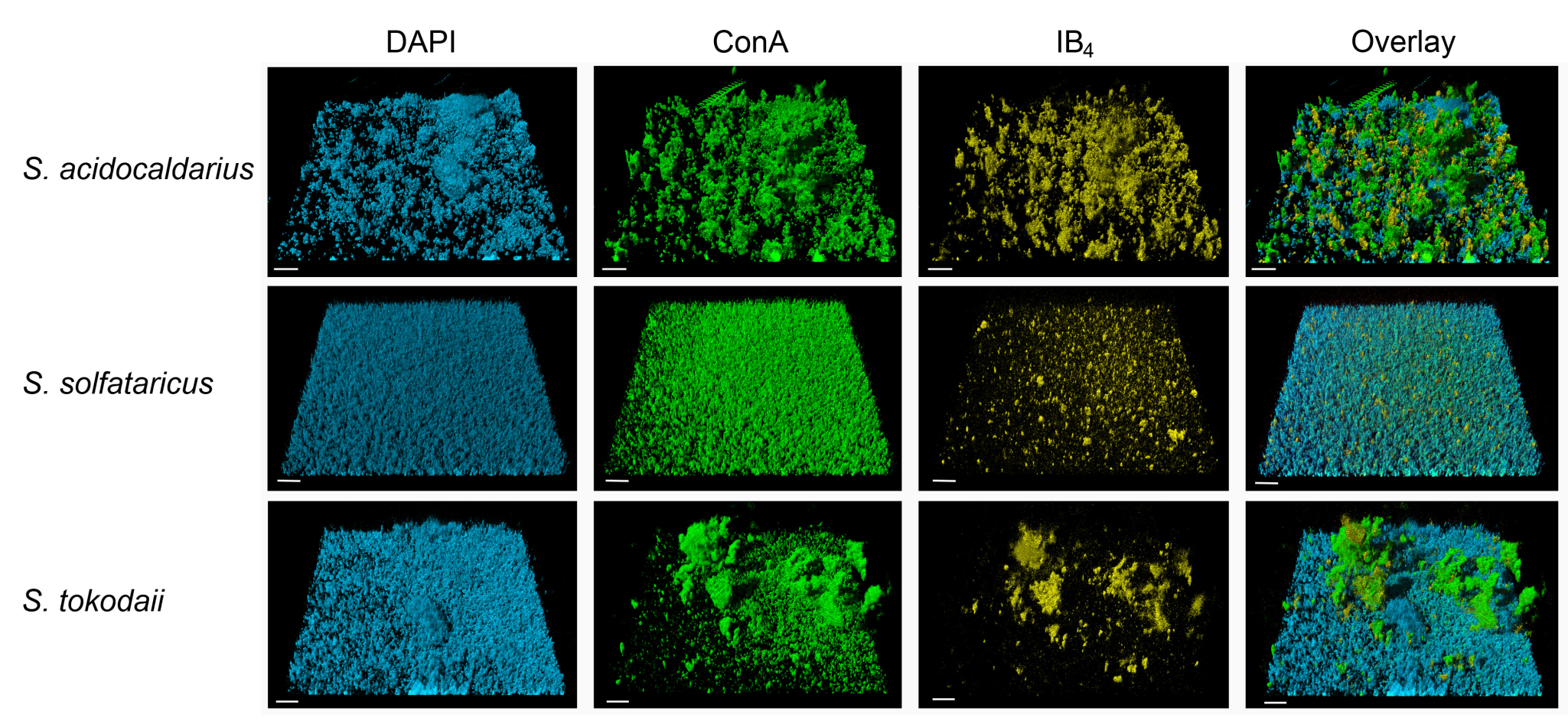

Supplement: Figure S3 — CSLM analysis of three day old static biofilms of S. acidocaldarius, S. solfataricus and S. tokodaii by lectins. After three days the biofilms of S. acidocaldarius (first row), S. solfataricus (second row) and S. tokodaii (last row) were incubated with DAPI and different lectins and were analyzed by CSLM. The first column shows the DAPI signal (blue), the second column the Con A signal (green), the third column the IB4 signal (yellow) and the last column the overlay of the other three channels. Bars are 20 μm in length. CLSM: confocal laser scanning microscopy. (5.80 MB TIF) [file pone.0014104.s004.tif]

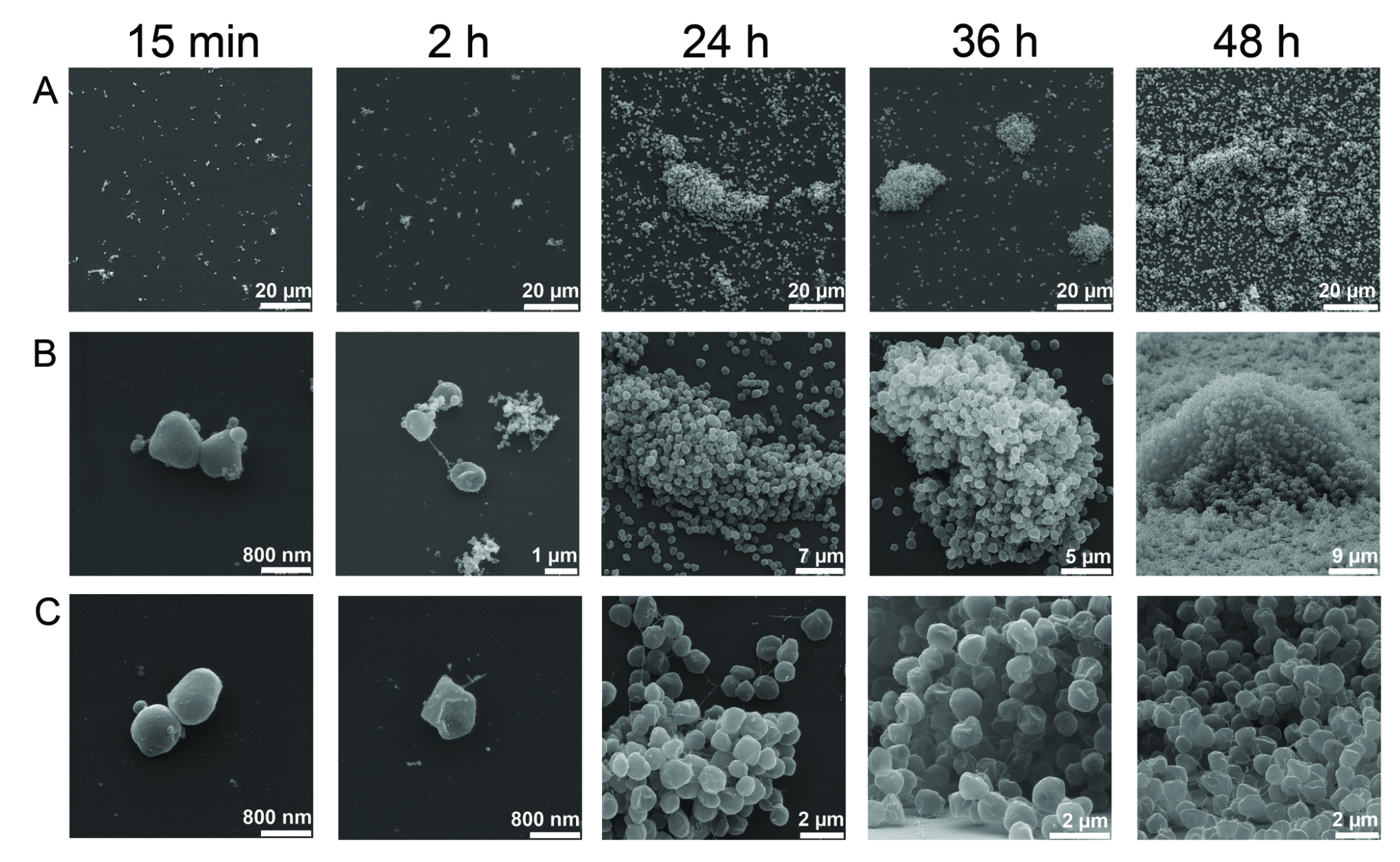

Supplement: Figure S4 — SEM pictures from early stages of S. acidocaldarius biofilm formation from 15 minutes to 48 hours after incubation. (A) shows the overviews and (B) and (C) more detailed views of the respective picture in A in the same column of the developing biofilms. The length of the bars is indicated in the images. SEM: scanning electron microscopy. (7.76 MB TIF) [file pone.0014104.s005.tif]

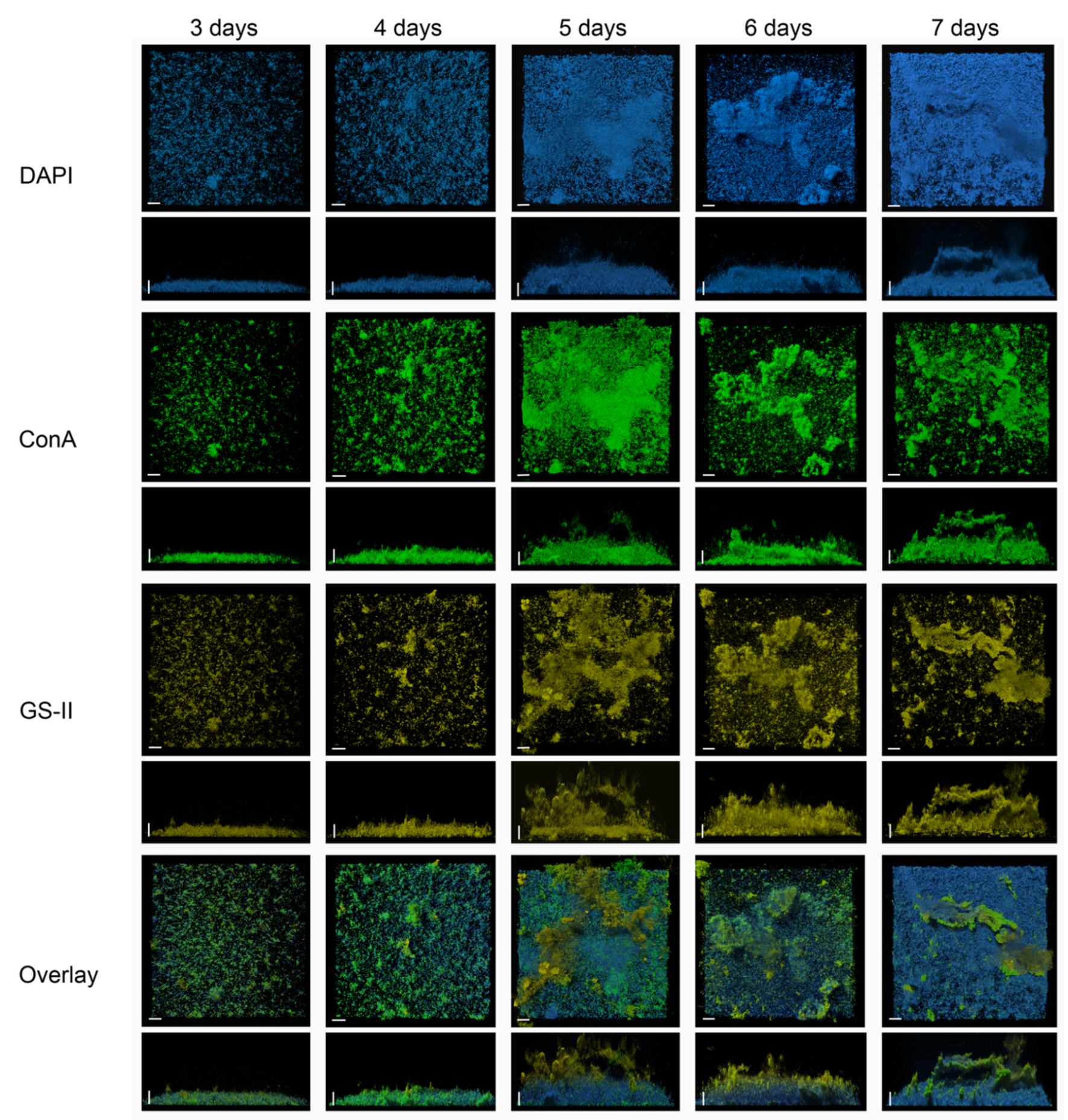

Supplement: Figure S5 — Lectin based analysis of developing static biofilm of S. acidocaldarius. Samples were treated with DAPI (blue channel), Con A (green channel) and GSII (yellow channel) and analyzed by CSLM. For each channel the top view and the side view is presented. Overlay shows all three channels again including top- and side views. Bars are 20 μm in length. (9.90 MB TIF) [file pone.0014104.s006.tif]
